# Iron, neuro‐bioavailability and depression

**DOI:** 10.1002/jha2.321

**Published:** 2021-12-05

**Authors:** Christian Berthou, Jean Paul Iliou, Denis Barba

**Affiliations:** ^1^ Department of Immuno‐Hematology INSERM UMR 12 27 LBAI University Brest Brest France; ^2^ Department of Psychiatry Pont Labbé Hospital France; ^3^ Health and Medical Center Le Guilvinec France

**Keywords:** deficiency, depression, iron, neuro‐availability

## Abstract

Medical management of iron deficiency (ID) requires to consider its consequences in biochemical and physiological plural functions, beyond heme/hemoglobin disrupted synthesis. Fatigue, muscle weakness, reduced exercise capacity, changes in thymia and modified emotional behaviors are the commonest symptoms integrated in the history of ID, dependent or not of the hemoglobin concentration. The relationship between depression and absolute ID (AID) is a condition which is often unrecognized. Neuro‐bioavailability and brain capture of blood iron are necessary for an appropriate synthesis of neurotransmitters (serotonin, dopamine, noradrenaline). These neurotransmitters, involved in emotional behaviors, depend on neuron aromatic hydoxylases functioning with iron as essential cofactor. Noradrenaline also has impact on neuroplasticity via brain‐derived neurotrophic factor (BDNF), which is key for prefrontal and hippocampus neurons playing a role in depression. Establishing the formal relationship between depression and AID remains difficult. Intracerebral reduced iron is still hard to quantify by neuroimaging and single‐photon emission computed tomography (SPECT) now tends to explore the neurotransmission pathways. AID has to be looked for and identified in the context of depression, major episode or resistant to conventional treatment such as serotonin reuptake inhibitor, and even in the absence of anemia, microcytosis or hypochromia (non‐anemic ID). Confronted to brain imaging, blood iron status evaluation is indicated, especially in depressed, treatment‐resistant, iron‐deficient young women. In patients suffering from depression, increase in the prevalence of AID should be considered, in order to deliver a suitable treatment, considering both anti‐depressive program and iron supplementation if AID.

## 1 INTRODUCTION

Over the last years, there has been a growing interest in the scientific community for iron metabolism and its implication in psychiatric disorders, notably due to the development of research in neurosciences. Discovery and understanding of iron homeostasis and iron body distribution, particularly in the central nervous system (CNS), raise many questions concerning the role of iron in neurodevelopment and in the CNS, its implication in psychiatric and neurological pathologies. A possible relation between iron deficiency (ID) and depression has to be more defined, explored and confirmed because these two entities constitute major public health problems around the world. ID is the most common micronutrient deficiency around the world and is the only one that is also found in industrialized countries, mainly in young women and children. According to the World Health Organization (WHO), depression affects 300 million people worldwide and is said to be twice as frequent in women as in men.

A disease has been long described, which was prevalent in young women and left patients with a lack of energy, shortness of breath and a greenish complexion. It was not until the 17th century that the term “chlorosis” was attributed to it (also known as “morbus virgineus”, “disease of young girls” or “pale colors”). This pathology, which is none other than ID anemia, was attributed to sexual or nervous disorders (“hysteria”). At that time, such traits were observed almost exclusively in women. Armand Trousseau reported among the symptoms of chlorosis: “nervous state, hysteria, melancholy, fickleness, muscular debility” [1]. This idea of “nervous disorders” as the cause of chlorosis lasted until the 20th century, even if some doctors of the 19th century already put forward the hypothesis of a “blood disease”. In the collective imagination, iron, being associated with Mars, God of war and symbol of strength, would therefore restore strength to weakened blood. Through the example of chlorosis, a link between psychological disorder and ID has been established. Trousseau specified that “these nervous disorders give way easily to martial preparations”.

The causes of depression include biological, genetic, psychological and psychosocial or environmental factors. According to the classical theory of depression, it is linked to a deficiency in monoamines (serotonin, dopamine (DA), noradrenaline (NA)). Indeed, these neurotransmitters (NTs) participate greatly in different aspects of emotional behavior.

The role of iron in neurotransmission is not sufficiently considered by practitioners. Iron is the cofactor of aromatic amino acid hydroxylases (phenylalanine, tyrosine, tryptophan (Trp)). Activity of these enzymes is the limiting step in the synthesis of DA, serotonin and indirectly norepinephrine. The latter would promote the production of the brain‐derived neurotrophic factor (BDNF). The balance of these three NTs determines the depressive syndrome.

In severe depression, cytokine‐mediated neuroinflammation will help to further deregulate the metabolism of NTs, directly and indirectly, through hyperexpression of hepcidin which induces brain functional iron deficiency (FID). The chronicity of the mental illness and depression will induce deleterious neuroinflammation and chronic low‐grade inflammation.

## 2 IRON DEFICIENCY

The prevalence of ID is difficult to assess precisely because there is low reliable epidemiological data on the subject, with mixed related pathological entities such as anemia, iron deficiency anemia (IDA) and isolated ID, without anemia (non‐anemic ID). Depending on the WHO estimates or meta‐analyses of population studies, the prevalence of ID is, respectively, 50% and 37% in women (42% and 25% in children), with large disparities depending on age, gender and regions of the world [2, 3]. According to the WHO, about one‐third of non‐pregnant women suffer from anemia worldwide and 40% of pregnant women [4]. In about 50% of cases, this is due to severe ID.

### 2.1 While erythroblast is an iron‐hungry bone marrow cell, a fair split in iron is needed among tissues

About 400 genes (about 2% of the human genome) are involved in iron metabolism and its homeostasis. Plurality of symptoms is related to plurality of body iron functions, involving and interacting with many proteins, thus making its physiology complex. Iron is involved in the transport of oxygen as a component of hemoglobin (Hb) and plays an essential role in erythropoiesis. Iron also participates in cell metabolism, energy production and electron transport (especially in mitochondrial muscle), cell respiration and proliferation but also differentiation, synthesis of nucleic acids and proteins and regulation of gene expression. It is also involved as a cofactor in many enzymatic reactions such as synthesis of catecholamines, free Trp leading to serotonin synthesis, phenylalanine and tyrosine metabolism leading to DA and NA components, synthesis of nucleotides and thyroid hormone metabolism, via the thyroid peroxydase.

Consequently, if heme/Hb reduced synthesis with anemia is the most well‐recognized consequence of ID, this condition also disturbed biochemical iron‐dependent functions in non‐erythropoietic cells, such as synthesis of myoglobin, cytochromes and brain hydroxylases. This led to non‐erythropoietic symptoms related to altered muscle mitochondrial function and electron transport chain, brain cell metabolism, neurotransmission and neuroplasticity [5].

Absolute iron deficiency (AID) [6] is a clinico‐biological condition which associates a decrease in total body iron stores (ferritinemia <30–50 μg/L, excluding a chronic inflammatory syndrome and hepatic cytolysis). AID can stand alone as non‐anemic ID, a clinical condition with isolated AID (I‐AID). Later, iron‐deficient erythropoiesis can trigger hypochromia (mean corpuscular Hb, MCH < 27 pg), then microcytosis (mean corpuscular volume, MCV < 82 fL) and eventually ends to anemia characterizing IDA (reactive thrombocytosis possible) [6].

AID can be asymptomatic but is often responsible for a clinical picture, associating the following symptoms and signs: skin and mucous pallor if anemia; chronic fatigue or even exhaustion; shortness of breath, exercise or resting tachycardia; impaired physical exercise performance related to muscle weakness; neuro‐sensory symptoms such as dizziness and/or tinnitus and/or phosphenes, headache, faintness, loss of consciousness or fall in the elderly; pagophagia, pathological craving for ice (or other forms of pica); also restless legs syndrome [1, 5, 7]. At worst, in case of severe anemia, angina can be induced by ischemic heart disease or heart failure (HF). Skin and mucous membrane disorders can be observed: pruritus, alopecia, koilonychia, brittle nails, dry hair or skin, “perlèche”, atrophic glossitis. In AID, a special attention should be paid in some situations such as pro‐ and post‐operative periods or pregnancy and postpartum.

Whatever the clinical context of AID, psychological and/or neuro‐psychological symptoms are common [1] but often little considered by healthcare professionals. There are sometimes referred as brain fog and/or “mummy brain” [1] such as a decrease in cognitive functions, feeling tired, learning and concentration disorders, reduced concentration, inability to focus and to think clearly, depression, anxiety and sleep disorder. The prevalence of these neuro‐psychological symptoms led us to consider the brain mechanisms in place to efficiently conserve its iron and the cerebral metabolic consequences of AID.

### 2.2 Isolated absolute iron deficiency, without anemia, as a clinico‐biological entity to consider

Since 2004, the WHO has identified non‐anemic ID as a public health problem. This concept of I‐AID or “absolute iron deficiency syndrome” was defined by Beutler et al. [8]. He demonstrated, in a randomized placebo trial, that women with ID without anemia, who were symptomatic (dizziness, fatigue), saw their symptoms improve under iron repletion. Biologically, I‐AID is defined by a normal Hb level and a serum ferritin <30 μg/L. Other studies support the definition of AID for a higher serum ferritin threshold (<50 μg/L) [9], inside which iron supplementation often corrects all or part of the symptoms found [10, 11].

Hypochromic microcytic anemia has to be considered as an end‐organ dysfunction of AID‐dependent erythropoiesis. AID with IDA or without anemia (I‐AID if severe, ferritinemia <20 μg/L with recurrent or risk of gastrointestinal malignancy) both require a rigorous etiologic research:

1. By upper and lower gastrointestinal endoscopy, search of distilling (may be occult) digestive hemorrhage (gastric ulcers, polyps, cancer; duodenal ulcers; colon cancer, colonic polyps, angiodysplasia, diverticular bleeding, etc.), autoimmune gastritis, *Helicobacter pylori* gastric infection and duodenal malabsorption, such as coeliac disease and its appropriate serological tests (transglutaminase IgA, i.e., IgA‐TTC antibodies, combined with IgA concentration, anti‐endomysial antibodies). Endoscopy leads to the realization of regular biopsies in three sites (fundus, gastric antrum and duodenum), always performed, looking for *H pylori* and duodenal villous atrophy, together with appropriate localized biopsies if mucous lesion(s) is (are) identified, for anatomo‐pathology analysis.
2. By a clinical (context of menorrhagia or meno‐metrorrhagia), gynecological imaging and/or uterine endoscopy, search for a gynecological lesion, functional hormonal bleeding (premenopausal women and girls) or gynecological bleeding favored by a primary hemostasis defect (von Willebrand disease, platelet dysfunction, carrier for hemophilia A or B, etc.).
3. All these conditions could be amplified by deficient nutrition (iron‐poor diet), inadequate stomach acidification (long‐term use of proton pump inhibitors or antiacids, atrophic gastritis, *H pylori*, after gastric bypass), in patients with pica (addictive ingestion of non‐nutritive foods, such as clay, soil, ice, etc.).

If unexplained IDA or unexplained severe I‐AID, one has to consider a video capsule enteroscopy for small intestinal bleeding lesion and a clinical skin examination for scars, observed on the upper limbs in the Lasthénie de Ferjol syndrome, in case of Münchhausen syndrome (self‐induced blood loss).

## 3 INTRACEREBRAL IRON METABOLISM

### 3.1 Blood–brain barrier as a multicellular complex regulating iron entry into cerebral interstitial fluid

Iron enters the CNS through the blood–brain barrier (BBB), which regulates iron CNS as an entry lock. The BBB protects the brain from a massive influx of iron. As other organs, brain needs a fine regulation of iron metabolism for optimal cell functioning. A disorder of iron regulation would lead to local iron accumulation, producing free radicals, oxidative stress and cell death (“ferroptosis”).

**FIGURE 1:**
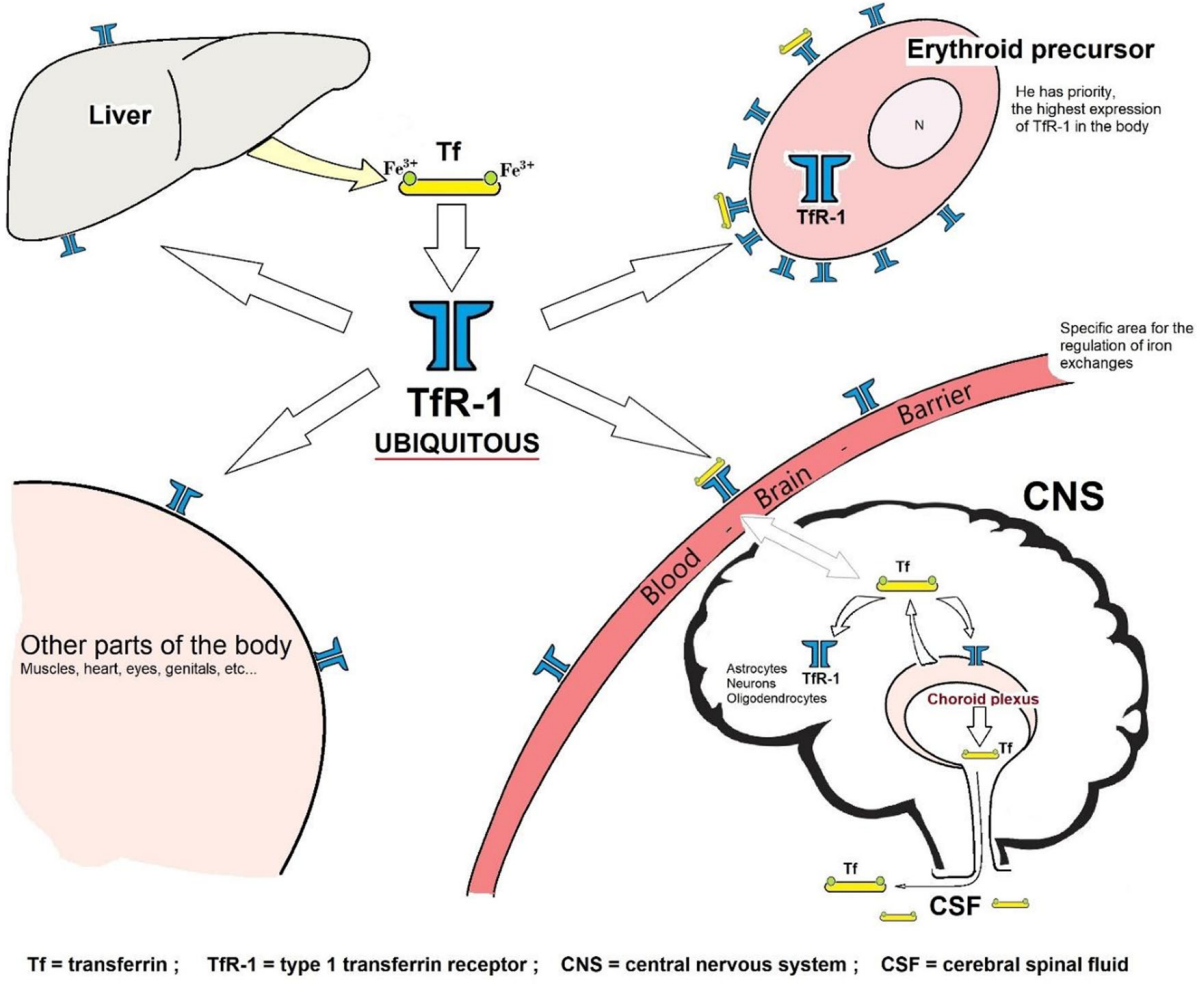
Schematic representation of the body distribution of the type 1 transferrin receptor (TfR1). In the central nervous system (CNS), endothelial cells of the blood–brain barrier (BBB) expressed a great density of the TfR1, just after erythroid precursors did and above other parts of the body. Oligodendrocytes and choroid plexuses‐dependent local transferrin added, in brain, iron transport provided by plasma transferrin synthetized by hepatocytes. Choroid plexuses secrete transferrin in cerebrospinal fluid (CSF). Brain transferrin can capture Fe^3+^ in interstitial fluid, thanks to ceruloplasmin ferrous iron oxidation

Plasmatic iron transport is provided by serum transferrin which is synthetized by hepatocytes. The transferrin receptor 1 (TfR1) is present in all body cells to facilitate the import of iron, except for mature red blood cells. TfR1 expressing cells internalize the complex “diferric Fe^3+^‐transferrin‐TfR1” in endosome which is able to release ferric Fe^3+^ ion from the complex by acidification and to reduce it to ferrous Fe^2+^ ion by STEAP3 enzyme. Fe^2+^ ion then crosses the endosomal membrane thanks to DMT1. In the body, the greatest density of TfR1 is found in the erythroid precursors (Figure 1).

In the CNS, it is endothelial cells of the BBB vessels that express it the most. In case of ID, there is an overexpression of the transferrin receptors and an increase in their density, induced by the HIF‐1 protein (stimulated by hypoxia), which interacts with the gene encoding TfR1 [12]. TfR1 expressed luminal endothelial cell, the cellular brain “entrance gate” for systemic differic iron Fer^3+^ plasma transferrin, is regulated by CNS iron status. Its expression is appropriately increased, for further serum iron uptake, in case of CNS ID [13], although the CNS iron sensing cell(s) remain(s) to be identified.

**FIGURE 2:**
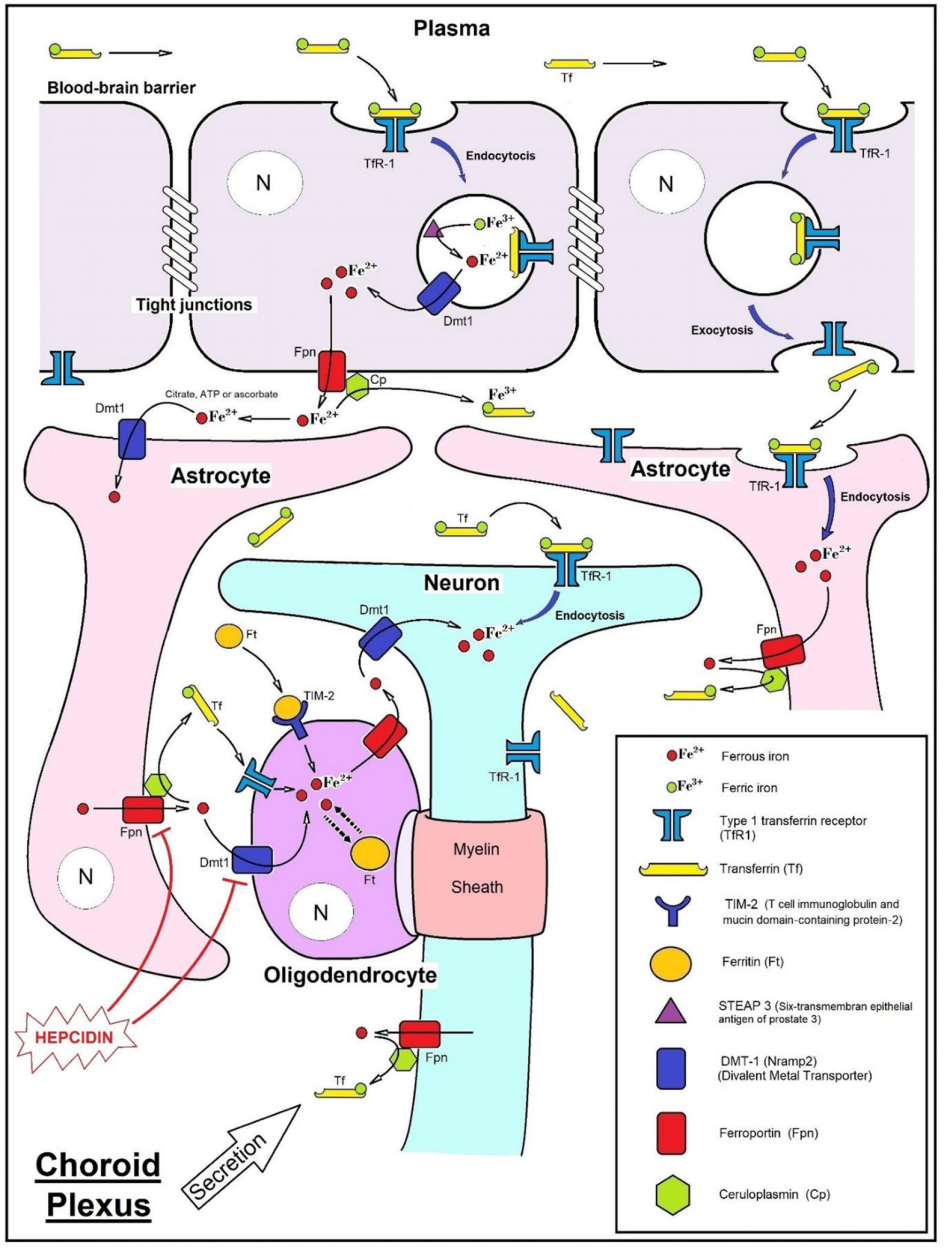
Intracerebral metabolism of iron and its neuro‐bioavailability. Brain endothelial cells propose two pathways for brain iron distribution from blood: a facilitated transporter pathway and a transcytosis pathway. The facilitated transporting pathway is linked to the ability of endothelial cells to reduce Fe^3+^ to Fe^2+^ within the vacuole, via acidification and STEAP3 protein. Ferrous iron Fe^2+^ uses transmembrane transporters to reach the brain interstitial fluid: DMT1, to get out the vacuole and export Fe^2+^ to the cytosol; ferroportin leading to appropriate Fe^2+^ increased in the brain interstitium, so as to make iron available to the astrocyte or to be re‐oxydated to Fe^3+^ due to the ceruloplasmin activity. The transcytosis pathway implicates endocytosis and exocytosis of the complex transferrin receptor (TfR1)‐Fe^3+^‐bound transferrin, from the luminal pole to the cerebral extracellular matrix (ECM) and interstitial fluid

Vessel endothelial cells capture, at their luminal pole, circulating iron Fe^3+^‐bound transferrin, via the TfR1 receptor, which forms an endocytosis vacuole (Figure 2). Endothelial cells can reduce Fer^3+^ to Fer^2+^ into the endocytosis vacuoles, via STEAP3 protein. Fer^2+^ uses the vacuole membrane iron transporter, DMT1, to reach cytoplasm and is then exported to the extracellular matrix (ECM) and interstitial fluid, via ferroportin (the cellular iron exporter coupled with ceruloplasmin). Ceruloplasmin is synthesized by the liver and is a ferroxidase of importance, because it is coupled to ferroportin throughout the body. In brain, astrocytes, just like hepatocytes in the liver, have the ability to secrete ceruloplasmin, a major ferroxidase protein which can turn ferrous iron Fe^2+^ into ferric iron Fe^3+^. Ceruloplasmin is concentrated on end‐foot processes of astrocytes by a glycophosphoinositide (GPI)‐dependent membrane anchoring. Astrocytic foot processes are arranged in close proximity with endothelial cell basal lamina and pericytes in the BBB [13].

Within the brain interstitium, exported iron ECM Fer^2+^ can either become available for astrocyte, oligodendrocyte and neuronal incorporation, through DMT1 or can be targeted to brain free transferrin in interstitial fluid, after ceruloplasmin oxidization to ferric iron Fe^3+^ (Figure 2). Note that transferrin is not only a plasmatic protein. Oligodendrocytes and choroid plexuses synthetized free transferrin, and choroid plexuses secrete it. Both are able to capture new Fer^3+^ after ECM Fer^2+^ ceruloplasmin iron oxidation [14]. In the choroid plexuses, the activation of serotonin 5‐HT2c receptors would lead to an increase in the production of transferrin. Serotoninergic neurotransmission influences the level of iron in the midbrain, on the one hand, via the action of the presynaptic serotonin transporter (SERT), and on the other hand, via the increase in the production of transferrin by the choroid plexuses after stimulation of the 5‐HT2c receptors.

Brain transferrin concentrations remain at around 10% of serum transferrin. This could explain that brain transferrin is available only for brain tissue as it cannot return to the systemic circulation [13]. Such a low level of brain transferrin also implicates that potentially damaging brain non‐transferrin‐bound iron (NTBI) may exist. Concentration of transferrin in the cerebrospinal fluid (CSF) and measurement of transferrin saturation in CSF could be a good reflection of the availability of iron in the brain and for better evaluation of brain iron status.

### 3.2 Oligodendrocyte‐neuron cell couple as the target of cerebral iron homeostasis

In the neuroglia, oligodendrocytes, the myelin initiating cells, are the most “iron‐hungry” cells in brain tissue. They can capture iron by three means: TfR1, DMT1 and by direct uptake of ferritin, in transit through the ECM, using the TIM receptor‐2 (Figure 2). Oligodendrocytes contain the majority of intracerebral iron and ensure the production of myelin sheathes around axons. Iron is directly involved in this production, as a cofactor necessary for the biosynthesis of cholesterol and lipids in the sheath.

For neuronal iron availability, astrocyte and oligodendrocyte export their Fe^2+^ via ferroportin, the only export protein of cellular iron, present in all brain cells. To capture it, DMT1 is highly expressed on neurons. Furthermore, by TfR1‐dependent neuronal endocytosis, neuronal processes take a source of iron‐differic Fe^3+^ transferrin from CNS interstitial fluid. CNS iron is stored mainly in the form of ferritin (except in the substantia nigra and in the locus coeruleus where there is a true “iron sink” protein called neuromelanin which stores it). Ferritin which is present in the axons and neurons could release ferrous iron from the neuronal cell body to the synapse, thanks to ferroportin present in synaptic vesicles.

In ID, iron acquisition by astrocytes and oligodendrocytes is reduced. Finally, ID induces a significant decrease in the intracerebral iron concentration [15, 16, 17, 18]. The neuronal consequence of FID is poorly defined. One can expect that, in chronic inflammatory situations leading to FID, like another body sites, the only cellular iron‐export protein that is ferroportin is down‐regulated by hepcidin, an inflammatory protein that decreases iron neuronal‐bioavailability [19]. ID is involved in disorders of synaptogenesis and myelinogenesis [20], disrupting the neurodevelopment of the child [21]. On the contrary, iron accumulation in the substantia nigra, due to the aging process, may contribute to the risk of neurodegenerative diseases (such as Parkinson and Alzheimer diseases) [22, 23].

### 3.3 Brain tissue specific skills in iron distribution

Transferrin can circulate through the ECM of the brain tissue. Free and intact transferrin in interstitial fluid can influence the brain redistribution of iron, as observed in hypotransferrinemic mice [13, 24]. Parts of brain tissue have developed specific skills in iron's stores management and make the iron distribution in brain unequal. The CNS contains 30–40 mg of iron (less than 1% of the body's total iron). In the substantia nigra and the locus coeruleus, production of neuromelanin provides an alternative to ferritin for storage and significantly increases the iron concentration in these specialized regions of the brain. High brain iron concentration is observed into the globus pallidus, followed by the putamen, the substantia nigra and the caudate nuclei. These structures, like the substantia nigra, are all parts of the basal ganglia.

In addition to their role in facilitating movement, the basal ganglia, are also presumed to have a role in emotional processes, behaviors and motivation [25]. For brain cells, as for other cells of human body, iron is involved in many common biological processes such as oxygen transport (O2), use of O2 by the mitochondrial chain and its citric acid cycle, synthesis of nucleic acids. Iron implication as cofactor in heme‐containing and non‐heme iron‐containing enzymes leads to disturbances in mitochondrial electron transport chain and Adenosine‐TriphosPhate (ATP) production [26]. Citrate, ATP and ascorbate influence DMT1 function.

Iron is also involved in the synthesis of myelin and the metabolism of NTs and could play an important role in emotional behaviors and neuroplasticity.

## 4 IRON DEFICIENCY, ALTERED NEUROTRANSMISSION AND DEPRESSION

According to the “classic” theory of depression, there is a deficiency in brain monoamines (serotonin, DA, NA), explaining why the main anti‐depressants used today are reuptake inhibitors (RIs) of these NTs, with the most common one being the serotonin RIs (SRIs). Their goal is to increase the concentration of these NTs intrasynaptically: an increased synaptic 5‐hydroxytryptamine (5‐HT) results in anti‐depressant effects.

**FIGURE 3:**
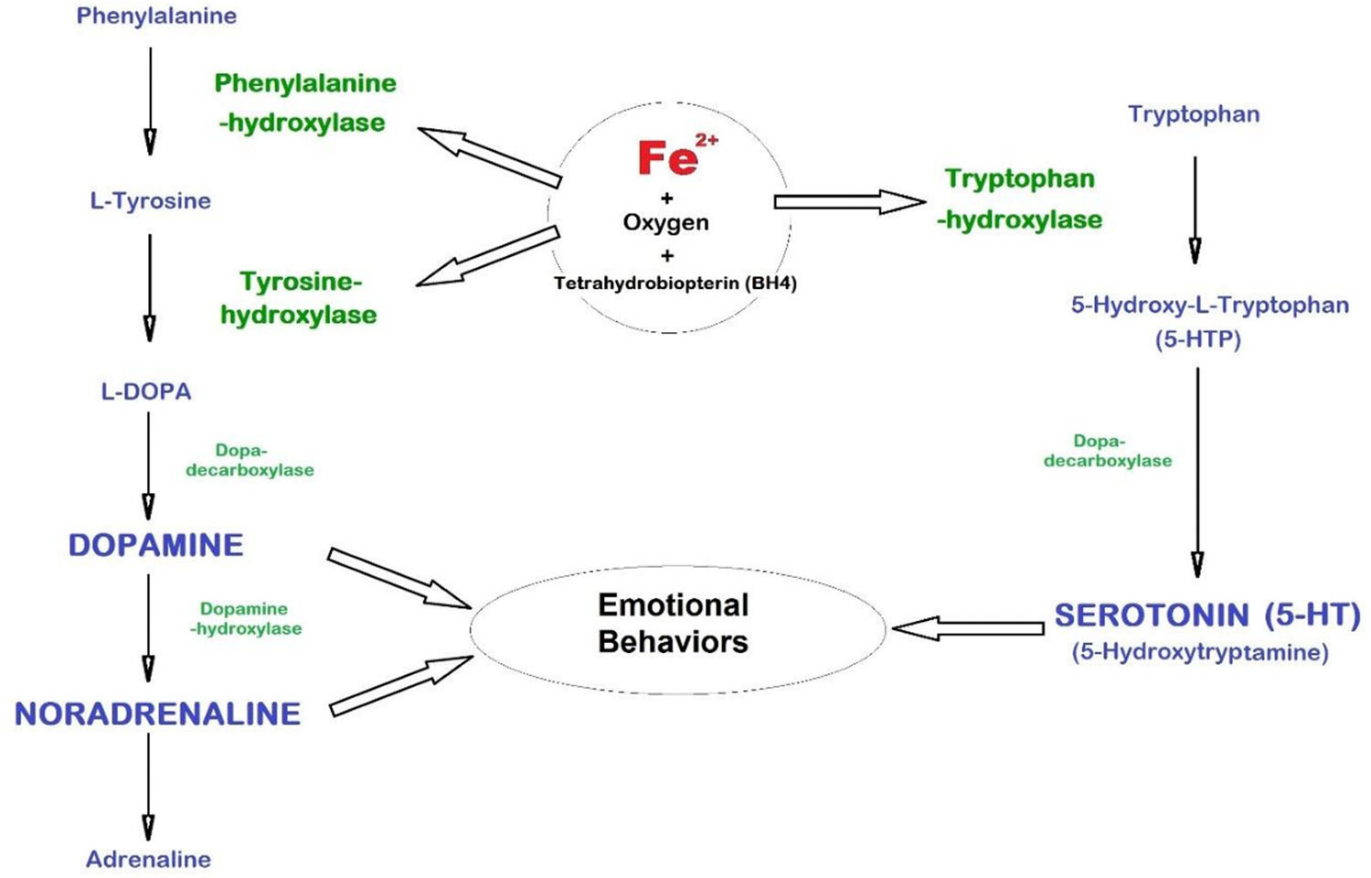
Iron implication in neurotransmitter production and function. Serotonin plays an important role in changes in emotional state. It is particularly involved in the management of moods, is associated with the state of happiness and leading the individual to maintain an emotional situation that is favorable to him. Dopamine is the main neurotransmitter of the motivational component of reward and reinforcement. Synthesis of serotonin and dopamine is dependent on brain hydroxylases which use iron and tetrahydrobiopterin (BH4) as cofactors. Their synthesis is hampered and deficient in case of iron deficiency

Fe^2+^ and tetrahydrobiopterin (BH4) are obligatory cofactors for the aromatic acid hydroxylases. These are the following enzymes, phenylalanine, tyrosine and Trp hydroxylases, acting together with O2. These three hydroxylases are needed for DA, NA, 5‐HT or serotonin synthesis (Figure 3).

### 4.1 A dangerous link between reduced neuronal iron and tryptophan availabilities

In depression, intracerebral neuroinflammatory mechanisms lead to an increase in body levels of pro‐inflammatory interleukins and cytokines (IL‐1β, IL‐6, TNF‐α, IFN‐γ) [19]. It should be noted that neuroinflammation is more an endogenous brain process than a systemic disease, is a chronic condition, taking place in a slow‐damaging inflammatory process.

These pro‐inflammatory factors induce the following brain consequences:

1. increased secretion of the master regulator of iron homeostasis, hepcidin, which freezes iron into astrocytes and microglia, inducing FID;
2. BH4 deficiency which, along with iron, is the cofactor of phenylalanine, tyrosine and Trp hydroxylases. BH4 deficiency reduces brain monoamines synthesis and an efficient DA, NA but also 5‐HT productions. This BH4 decrease is due to guanosine triphosphate (GTP) cyclohydrase enzyme induction, which consequently promotes neopterin synthesis. Neopterin increases oxidative stress and inhibits the proliferation of erythroblastic precursors;
3. increased Trp systemic degradation, reduced plasmatic Trp level, alongside with diversion of Trp metabolism, foster the kynurenine (Kyn) pathway [27]. This process is due to indoleamine 2,3‐dioxygenase (IDO1 and IDO2) enzyme induction. Cytokine‐mediated neuroinflammation induces microglia transformation of Kyn to the neurotoxic 3‐hydroxykynurenine (3‐HK) and quinolinic acid (QUIN) metabolites. QUIN increases transcription of hepcidin and both with 3‐HK, favor HIF degradation which reduces erythropoietin (EPO) synthesis. QUIN and 3‐HK increase oxidative stress. QUIN is a potent agonist of the N‐Methyl‐D‐Aspartate (NMDA) Receptors in the glutamatergic pathway, resulting in neuro‐excitotoxicity.

Neuronal ID and reduced neuron Trp availability both determine low serotonin synapse concentration associated with the development of depression. The NTs serotonin, DA and NA participate in different aspects of emotional behavior. The balance of these three NTs conditions the depressive syndrome. Iron and BH4 are cofactors of aromatic amino acid hydroxylases governing enzymatic reactions, a critical and limiting step for the synthesis of NTs.

### 4.2 Iron deficiency and serotonin pathway dysregulation

Decreased circulating iron Fe^3+^‐bound transferrin and of free Trp concentration available to cross the BBB, both determine serotonin metabolism dysregulation and decreased serotonin concentration. Reduced synaptic connectivity at the level of the hippocampal and prefrontal cortex will trigger neuro‐behavioral manifestations, neuro‐psychological disorders and depression.

Trp hydroxylase is necessary for the synthesis of serotonin (5‐HT), via 5‐hydroxyl‐L‐tryptophan (5‐HTP) (Figure 3) [27]. More than 98% of the body's 5‐HT is present in platelets. Most of the secretion is provided by enterochromaffin cells in the intestine. Remaining 1% of 5‐HT, at the level of the CNS, is synthesized on site in the Raphe nuclei, from L‐tryptophan, which passes BBB, unlike 5‐HT. There are more than 15 serotonin receptor subtypes, grouped into seven families (5‐HT1, 5‐HT2, etc.), with various functions. Iron Fe^3+^‐bound transferrin and free Trp, both crossing the BBB, are crucial for serotonin formation and also, for biosynthesis of melatonin (N‐acetyl‐5‐methoxytryptamin or 5‐MT) synthesis, via N‐acetyl‐5‐HT [27].

Of note that, in the CNS, there is a positive feedback loop between serotonin anabolism and its capacity to stimulate iron encephalic entry, connecting serotoninergic transmission to iron transport. A study has shown that serotonin transporter SERT knockout (KO) mice had a decrease in iron in the midbrain and basal ganglia [28]. ID in rats resulted in a decrease in the intracerebral concentration of Trp and serotonin [16]. Of note that Thompson [29] suggested the involvement of 5‐HT in the intestinal absorption of iron.

### 4.3 Iron deficiency and dopamine pathway dysregulation

ID also impacts DA biology, which could be more damaging in infancy and young adults with alteration in the mesolimbic pathway [30]. Iron metabolism is also involved in dopaminergic pathway and neurotransmission. The substantia nigra could be particularly vulnerable to ID responsible for reduced tyrosine hydroxylase activity, leading to impaired DA synthesis which can cause psychological and motor problems in adults [31]. SERT bioactivity impacts dopaminergic signaling by its capacity to modulate intracerebral iron homeostasis. SERT‐dependent decrease in the concentration of intracerebral iron impacts dopaminergic (and noradrenergic) neurotransmission, by its action on the synthesis of DA (DA anabolism implicates the iron‐dependent phenylalanine and tyrosine hydroxylases) and by (reversible) decrease in the density of dopaminergic D2 receptors and presynaptic DA transporter (DAT), which ensures presynaptic recycling (Table 1) [32, 33].

**TABLE 1:** Diverse role of iron in neurotransmission and neurogenesis: neurotransmitters involved in emotional behavior and neuroplasticity

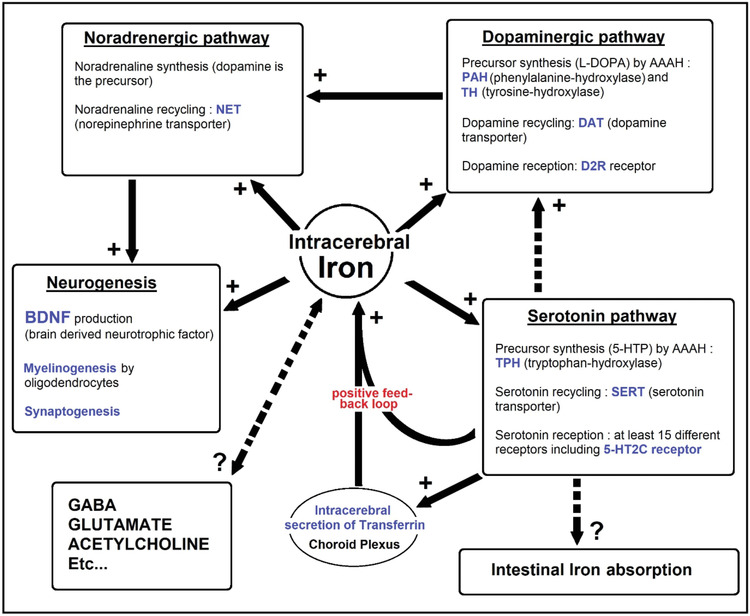

*Note*: Brain iron is essential in the process of neurotransmitter synthesis implicating the serotonin, dopaminergic, noradrenergic pathways and neuroplasticity. Dopamine is a noradrenaline precursor. Reduced noradrenaline synthesis leads to a deficiency in brain‐derived neurotrophic factor (BDNF). As a result, BDNF decreases with iron deficiency, leading to reduced neurogenesis and even hippocampus and prefrontal cortex atrophy that could play a role in depression.

DA is the precursor of NA which influences neurogenesis via the BDNF.

## 5 REDUCED BDNF AND NEUROPLASTICITY AS A CONSEQUENCE OF IRON DEFICIENCY

Depression can be thought as a combination of decreased synaptic connectivity and/or neuronal atrophy in the hippocampus and the prefrontal cortex.

Brain imaging by magnetic resonance imaging (MRI) has demonstrated changes in the volume of the frontal and prefrontal cortex and the hippocampus, associated with atrophy with loss of neuronal and/or glial cells. In the hippocampus, the decrease in volume would depend on the duration and severity of the depression, and could be prevented by drug treatments such as SRI. However, these changes would not be linked specifically to depression; they have also been described in post‐traumatic stress syndromes or schizophrenia.

Original studies have shown that these structural changes illustrate the concept of brain neuroplasticity, which is a formation of new neurons and reorganization of circuits in the brain, even in adulthood.

### 5.1 Brain iron is key for prefrontal and hippocampus neurons via BDNF

In the hippocampus zone (brain region involved in cognition and emotions), production of BDNF seems critical, as a factor essential for the proliferation, differentiation and survival of neurons [34]. BDNF decreases with ID, leading to atrophy of the hippocampus in rats [35, 36]. NA stimulates and increases BDNF production (Table 1). In cellular models, iron deprivation induces an increase in the degradation of the NA transporter Norepinephrin Transporter (NET). Inflammation‐related BDNF reduction leads to atrophy of the prefrontal cortex and the hippocampus. Iron may be essential for survival of hippocampus neurons and thus playing a role in depression.

### 5.2 Brain erythropoietin and BDNF

EPO, a growth factor for erythrocyte precursors in the bone marrow, also acts in the regulation and growth of a number of tissues, including cells of the nervous system. Thus, it plays the role of neuronal growth factor [37]. Its biosynthesis certainly takes place mainly by the renal cortex, but also in the brain parenchyma [38].

Experimental data in animals reveal the highest concentrations of EPO and EPO receptors in the choroid plexuses and the striatum; at a lower level, in the prefrontal cortex and the hippocampus. In animal models, it has been shown that EPO induces neurotrophic gene expressions, especially those encoding BDNF, in the prefrontal cortex and the hippocampus [38].

## 6 DEPRESSION

Depression, which is characterized by sadness and/or loss of pleasure, is a mental pathology that affects all age groups and all socio‐professional categories. In psychiatric pathology, we speak of a characterized depressive episode, formerly called major depressive episode. Its diagnosis is based on classifications, the reference for which in France is the WHO CIM‐10. Another, more complex classification is used clinically and for research purposes: DSM‐5.

Depression has health, economic and social repercussions. Depression is responsible, in France, for 35%–45% of work stoppage. Depression is associated with numerous somatic and psychological comorbidities (smoking, chronic alcoholism, reduction in physical activity), which accentuates its importance in terms of public health. The main risk of depression remains suicide. The risk of attempted suicide is multiplied by 21 in the event of a depressive episode [39].

The causes of depression include biological, psychological and psychosocial or environmental factors [40]. These parameters include behavior/well‐being factors, such as psychosocial stress/distress, anxiety, circadian rhythm, coping, pain; demographical ones, such as age, gender, social support; functional status, such as performance status, physical activity level. They all can influence development, severity and duration of depression. An associated family factor can be identified. A person whose parent has had depression is two to four times more likely to have one. There is therefore an individual susceptibility, which could be partly genetic in origin. Genetic variations associated with this vulnerability have been confirmed, in particular for the genes encoding the serotonin transporter SERT and BDNF proteins. In the CSF and serum of depressive patients, lower levels of BDNF have been found [41]. Recently, neurobiologists emphasized the important role of cytokines in continuous low‐grade inflammation related to a chronic mental disorder.

## 7 DEPRESSION, IRON DEFICIENCY AND CLINICAL STUDIES

In order to analyze the link between AID and depression, using the PRISMA quality criteria, we analyzed 25 articles published in the international literature. The majority of studies agree on establishing a link between ID and depression. Ten studies support an association between depression and iron status. Nine studies show an improvement in depressive symptoms after iron supplementation, either oral (per os administration, PO) or intravenous (IV) [10, 41, 42]. Only eight studies are randomized controlled clinical trials. The rest of the studies are made up of non‐randomized intervention studies, cross‐sectional studies and case–control studies. However, the majority of studies have used screening scales, which remain validated and reliable.

### 7.1 Iron deficiency as a risk factor for postpartum depression

Low blood Hb levels, iron status and low ferritinemia levels in the postpartum (but not during pregnancy) have all been associated with postpartum depression (PPD) [43, 44, 45]. In women with PPD, two randomized, controlled, double‐blind clinical trials, showed that early iron supplementation improved their symptoms of PPD, including women with postpartum IDA or I‐AID [46, 47]. In a similar way, in HF, if anxiety and depression are present, the symptoms disappear after iron IV treatment, together with improved ventricular function [48].

### 7.2 A definitive relationship between iron deficiency and depression?

About the relation between AID and higher psychological distress, there are weaknesses, linked to the nature of the studies. Most of the studies looked at populations either suffering from IDA and/or I‐AID, looking for depressive symptoms and/or improvement under iron supplementation. Very few studies (only six out of 25) had in their initial selection a population of depressed patients and in a second step, analyzed their martial balance (+/‐ supplement them).

### 7.3 A suitable iron status approach in depressed psychiatric patients

In order to use a psychiatrist to make the diagnosis of depression, according to the WHO CIM‐10 classification, we conducted a prospective and observational study, in collaboration with general practitioners, collecting a large cohort of depressed patients from a psychiatric clinic and a control group (290 and 126 patients, respectively) (Table 2). Patients were hospitalized for a characterized depression, either persistent despite treatment initiated on an outpatient basis or severe or resistant depression. Patients with a systemic inflammatory syndrome were excluded from the study.

**TABLE 2:** Average age and gender ratio of patients included in the study

	Depressed patients, *n* = 290	Control, *n* = 126	*p*
Average age (±standard deviation)	54.4 (±15)	58.4 (±15)	<0.05
Gender ratio, M/F (%)	108 (37.2)/182 (62.8)	57 (45.2)/69 (54.8)	0.125

Iron metabolism evaluation was analyzed using the following biomarkers (ferritinemia, transferrin saturation coefficient, implicitly including transferrinemia) and systematically integrated into the complete initial biological assessment. A significant increase in the prevalence of hypoferritinemia <50 μg/L (AID) was observed in the “depressed” group (26.6%  vs. 11.9%; *p* < 0.001). Surprisingly, a significant increase in the prevalence of hypotransferrinemia <2 g/L was observed in people with depression (21.7%  vs. 5.5%; *p* < 0.001). Hypotransferrinemia was independent of any protein loss, either of digestive or renal origins. Finally, the “depressed” group showed a prevalence of ID (absolute and functional related to hypotransferrinemia) of 48.3% against 17.5% in the “control” group (*p* < 0.001), so a prevalence almost tripled in the depressed patients (Table 3).

**TABLE 3:** Correlation between depression and iron deficiency

		Depressed patients, *n* = 290	Control, *n* = 126	*p*
Ferritinemia <50 μg/L and/or transferritinemia <2 g/L (%)	Yes	140 (48.3)	22 (17.5)	<0.001
	No	150 (51.7)	104 (82.5)	

The hypotransferrinemia we observed in the depressive population could correspond to a new form of FID. After iron capture by the BBB, it seems that intact transferrin in brain interstitial fluid is critical for appropriate distribution to the brain areas needing iron, which is not found in hypotransferrinemic mice [13, 24]. These data and their signification have to be confirmed by subsequent clinical studies. The correlation between ID and depression, through the notion of neuro‐bioavailability, is certainly stronger than supposed. Our work supports this hypothesis. The results indeed show an increase in the prevalence of ID in patients suffering from depression, favoring, in this context, regular blood iron metabolism assessment and serum transferrin dosage.

An increase in the prevalence of ID in severe depression must be confirmed by large controlled studies. Such clinical studies must be carried out in a psychiatric environment, in collaboration with the hematologist. Identification of the role of iron in depression or mood disorders will lead to deliver a suitable treatment, considering both anti‐depressive program and iron supplementation if ID or AID.

## 8 CEREBRAL IRON DEFICIENCY AND NEUROIMAGING: DON'T FORGET IT IN RESISTANT DEPRESSION

In front on any severe or prolonged depressive syndrome, MRI eliminates an organic process (brain injury or tumor) and it can provide information on change in the volume of the hippocampus and/or the prefrontal cortex.

To confirm the involvement of iron in depressive illness, it could be of interest to develop and/or extend the indications for brain imaging quantifying iron [49]. This type of imaging, based on MRI, in T2 and T2* sequence in particular, is mainly used in Parkinson's disease, where an accumulation of neurotoxic iron has been identified in certain regions of the brain, and in other neurological pathologies, such as Alzheimer's disease, multiple sclerosis, Wilson's disease, Huntington's disease. Brain imaging for quantification of ID remains a challenge.

The role of NTs in the pathogenesis of depression suggests the possible importance of neuroimaging in psychiatric pathology. We now have functional‐type imagery, which allows direct or indirect measurements of brain activity to be obtained. These methods correspond to single‐photon emission computed tomography (SPECT), positron emission tomography (PET) and functional MRI [50]. The most widely used nuclear imaging techniques are SPECT and PET‐Scan, which use short‐half‐life radioactive tracers.

Rapidly expanding SPECT imaging tends to explore neurotransmission pathways. Impaired dopaminergic transmission has been demonstrated in a small population size of 19 depressive individuals (anhedonia), showing that the binding of the DAT tracer was significantly lower in the basal ganglia (putamen, the caudate nuclei, the globus pallidus, the substantia nigra), in subjects with anhedonic depression, compared to healthy subjects [50]. Such results in depressive patients should, systematically, lead to body iron assessment, as a decrease in the concentration of intracerebral iron negatively impacts dopaminergic neurotransmission and concomitant decrease in DAT. Scientists are currently struggling to find reliable and valid radio‐tracers in humans, especially for the study of serotonergic transmission. Presently, the most widely used SPECT imaging in clinical practice is the DAT‐Scan, which provides information on neurodegenerative transmission such as in Parkinson disease.

## 9 IRON DEFICIENCY, DEPRESSION AND TREATMENT

### 9.1 AID needs symptomatic and etiologic treatment

Symptomatic treatment for ID is oral iron for at least 3 months until serum ferritin stably reach the level of, at least, ≥30 μg/L. Between 30 and 50 μg/L level of serum ferritin, debatable iron supplementation is of concern, since no international consensus is found. Nonetheless, we consider that a ferritinemia lower than 50 μg/L should benefit from iron compensation in support of the anti‐depressant treatment [9, 51].

Investigating the cause of ID is critical in order to treat a mucous lesion responsible for:

1. gastrointestinal (which can be occult) or gynecologic blood loss (fibroids or uterine cancer, etc.), also considering menstrual blood loss volume in premenopausal women and girls with a hormonal gynecological dysfunction and in young women using an intrauterine device;
2. intestinal mucosal dysfunctions: coeliac disease with decreased duodenal iron intake or inflammatory bowel diseases.

A condition favoring ID must be analyzed, such as iron‐poor diet, long‐term usage of proton pump inhibitor [52], an underlying congenital or acquired bleeding primary hemostasis disorder, aspirin and other non‐steroid anti‐inflammatory drugs (stomach erosions or ulcers), repeated blood donations, autoimmune gastritis, pregnancy after the second trimester [2, 47, 54].

A treatment failure (refractory AID) to go to normalization of blood parameters (Hb, MCV, MCH and finally ferritin level ≥30 μg/L) questions the oral iron treatment (non‐compliance, malabsorption), persistent (distilling or occult) blood loss, *H pylori* leading to atrophic or autoimmune gastritis (Biermer disease), or a mutation in the *TMPRSS6* gene with iron‐refractory IDA (IRIDA).

### 9.2 AID in depression: Oral (PO) or parenteral (IV) iron supplementation?

As AID, with or without anemia, is a source of symptoms including fatigue and depression, researchers compared the effectiveness of iron supplementation depending on the route of administration. Considering efficient duodenal absorption on iron supplementation, clinical recommendations go to oral intermediate doses and on alternate days in mild IDA, higher doses has to be considered in severe IDA or by parenteral iron therapy [54].

In women who had a postpartum hemorrhage (PPH) [47, 53, 54], they hypothesized that an IV iron injection within 48 h after childbirth would be more effective than daily administration of PO iron on symptoms of fatigue (primary endpoint) and depression (secondary endpoint). Of note that iron supplementation is critical in the postpartum period as this therapy has demonstrated its capacity to effectively decrease risk of PPD (which is not the case if given during pregnancy) [44]. For the two endpoints (fatigue and depression), multidimensional fatigue inventory (MFI) scale for fatigue, Edinburgh Post‐Natal Depression Scale (EPDS) scale for PPD, the mean scores decreased over time with a significant difference between the two groups in favor of IV iron. Indeed, the mean scores were significantly lower in women treated with IV iron 1–8 weeks after childbirth. At 12 weeks, this significant difference disappeared.

These studies emphasize the importance of parenteral iron compared to oral supplementation, according to the benefit of rapid iron recharge, in particular in psychiatry. Yet, in 2021, in France, iron IV administrations are still far too restrictive and not known enough. Obviously, in mental health, IV iron supplementation have to be considered in case of ferritinemia <50 μg/L, together with the anti‐depressive program.

### 9.3 A future place for EPO?

In humans, an improvement in mood was confirmed, in healthy subjects, 3 days after EPO administration, compared to subjects treated with placebo [55]. More recently, another study suggested an improvement in negative symptoms in patients treated EPO and suffering from uni‐ or bipolar depression [56]. It can therefore be assumed that an increase in EPO, by subcutaneous administration, decreases the synthesis of hepcidin via erythroferrone. Storage iron is then exported to plasma, as ferroportin inhibition stops.

In depression, action of EPO at the level of the CNS, together with iron compensation if AID, opens up avenues for further research in the near future as, in general, the role of EPO in neuroprotection [57].

## 10 CONCLUSION

AID is really a multifunctional disease, which is consistent with the human iron‐proteome [58, 59]. Exploration of iron metabolism in the CNS would be necessary for a good understanding of the relation between iron and neurodegenerative diseases, iron and the psychological status, iron and depression, continuous and chronic brain low‐grade inflammation [60] and iron metabolism. Anemic syndrome alone could impact cognitive and psychomotor functions in addition to the potential effects of I‐AID. Few “powerful” studies showed, with a high level of evidence, a formal link between depression and AID.

Investigators used to look for a depressive state in the context of AID, with or without anemia. It would be more relevant to also study iron metabolism in the context of characterized depressive state. In addition, in this context, MRI remains a reference for brain morphological analysis, in particular looking for a change in the volume of the prefrontal cortex and/or the hippocampus areas. Furthermore, this investigation eliminates a brain organic pathology. A PET‐Scan could be indicated in order to look for an atrophy of the prefrontal cortex and the hippocampus and/or a deficient vascularization. If resistant depression is observed, a DAT‐Scan investigation could confirm a deficient DA pathway and redirect treatment.

Blood, iron and systemic inflammatory biomarkers (CRP for acute and fibrinogenemia for chronic inflammation), beyond Hb, a full blood count with hemogram, a film with an erythrocyte smear, serum (or plasma) ferritin and fibrinogenemia assays should be included in any standard general medical assessment, especially in any initial assessment of a psychiatric patient. We have to consider the minimum values of ferritinemia to define ID (<30 μg/L or better <50 μg/L, without a chronic systemic inflammatory syndrome) [51, 53]. ID always leads to (a) determine a mucosal cause of distilling bleeding, even in occult digestive bleeding justifying endoscopic investigations and (b) prescribe iron supplementation (PO or even better IV), in order to obtain stable ferritinemia at least ≥30 μg/L or even 50 μg/L. We need to separate I‐AID and IDA, as a randomized placebo‐controlled trial demonstrated that iron‐related symptoms disappear under iron compensation, in the context of ID without anemia [8, 61].

## CONFLICT OF INTEREST

The authors declare no conflict of interest.
